# Achieving Precision Healthcare through Nanomedicine and Enhanced Model Systems

**DOI:** 10.1021/acsmaterialsau.3c00073

**Published:** 2023-12-18

**Authors:** Elin Svensson, Ula von Mentzer, Alexandra Stubelius

**Affiliations:** Division of Chemical Biology, Department of Life Sciences, Chalmers University of Technology, Gothenburg 412 96, Sweden

**Keywords:** Personalized Medicine, Precision Medicine, Nanomedicine, Drug Delivery, Model Systems, Vessel-on-a-Chip, Bioreactors, Joint Drug Delivery, Cartilage Transport

## Abstract

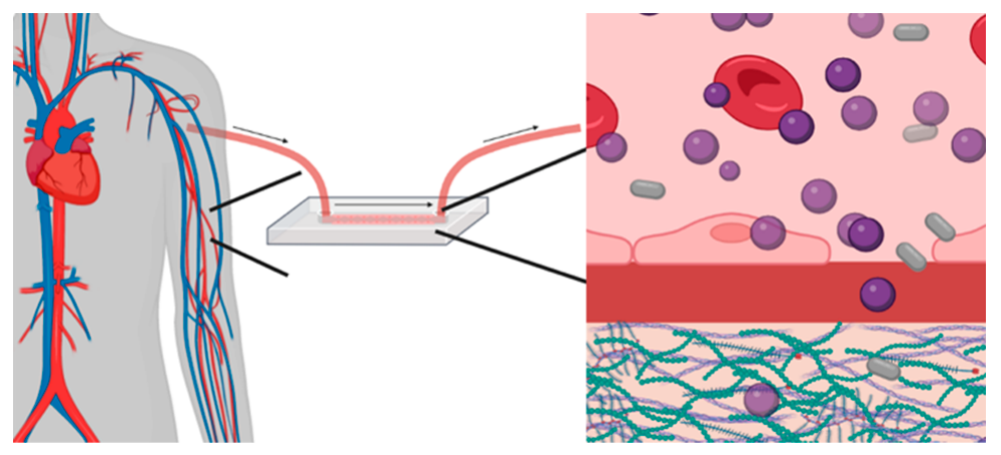

The ability to customize medical choices according to an individual’s genetic makeup and biomarker patterns marks a significant advancement toward overall improved healthcare for both individuals and society at large. By transitioning from the conventional one-size-fits-all approach to tailored treatments that can account for predispositions of different patient populations, nanomedicines can be customized to target the specific molecular underpinnings of a patient’s disease, thus mitigating the risk of collateral damage. However, for these systems to reach their full potential, our understanding of how nano-based therapeutics behave within the intricate human body is necessary. Effective drug administration to the targeted organ or pathological niche is dictated by properties such as nanocarrier (NC) size, shape, and targeting abilities, where understanding how NCs change their properties when they encounter biomolecules and phenomena such as shear stress in flow remains a major challenge. This Review specifically focuses on vessel-on-a-chip technology that can provide increased understanding of NC behavior in blood and summarizes the specialized environment of the joint to showcase advanced tissue models as approaches to address translational challenges. Compared to conventional cell studies or animal models, these advanced models can integrate patient material for full customization. Combining such models with nanomedicine can contribute to making personalized medicine achievable.

## Introduction

**Figure 1:**
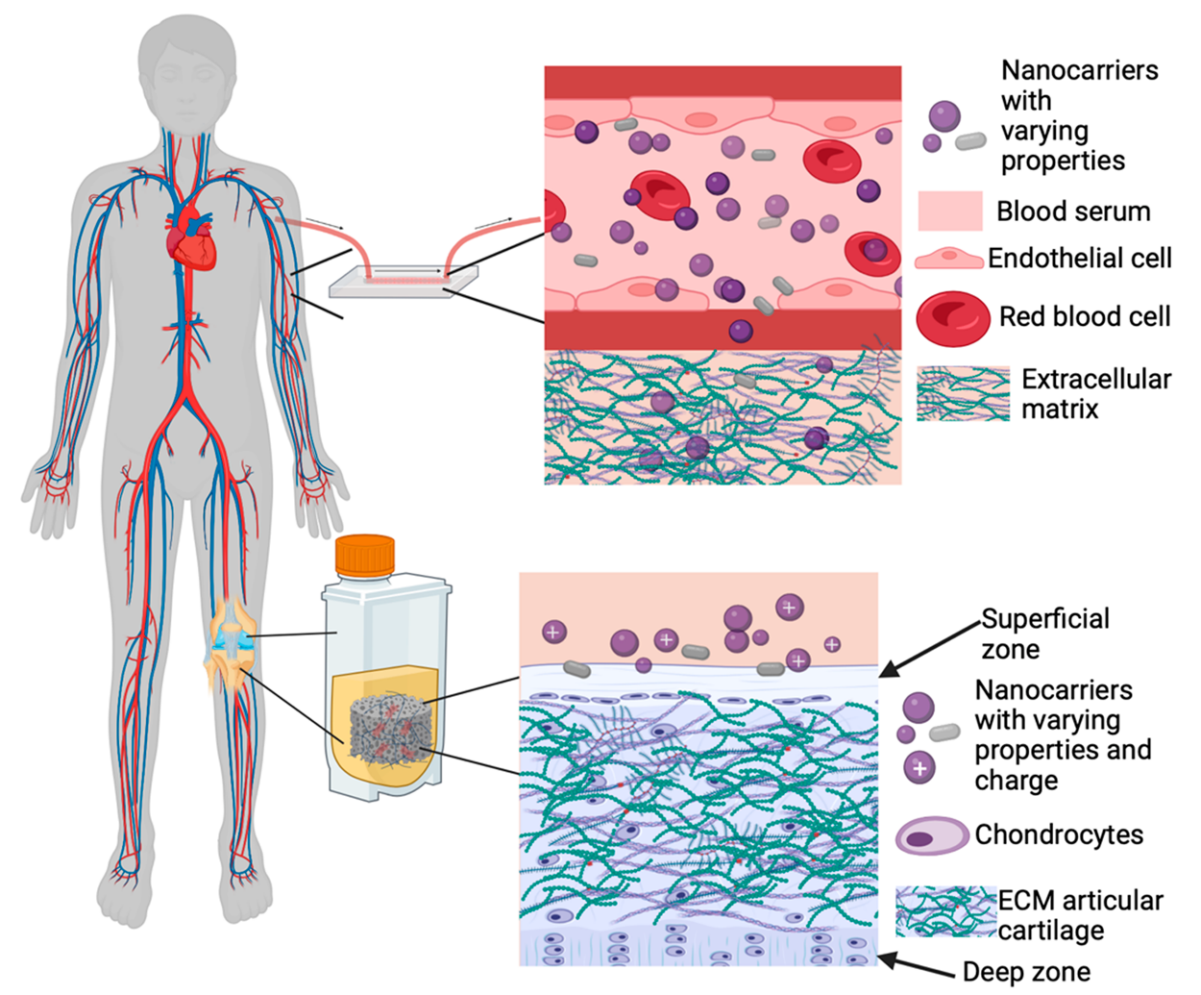
Models of NC delivery to target organs and cells. Delivery efficiency is dictated by the biological environment and the NC properties. Intravenous (i.v.) injection delivers NCs via the capillaries into the target organs, which can be modeled using vessels-on-a-chip. In the bloodstream, NCs are subjected to laminar flow, collisional and binding interactions with blood cells, and interactions with serum proteins. During delivery, NCs will interact with the endothelial cells, be taken up, and, in some cases, transcytosed to underlying tissue. Alternatively, NCs exit the blood via fenestrations (holes/pores) and can thus reach organ-specific cells. Intraarticular delivery to the joint can be modeled in advanced tissue systems, such as bioreactors. NCs to the chondrocyte can be challenging, and they have to be specifically engineered to avoid the rapid turnover of synovial fluid and enter the dense, negatively charged cartilage. Created with BioRender.com.

The paradigm shift toward personalized medicine, characterized by tailoring medical decisions based on individual genetics and biomarker profiles, has emerged as a groundbreaking approach in healthcare. To realize the full potential of personalized medicine that combines diagnostics, treatment approaches, and preventative actions, the International Consortium for Personalized Medicine (ICPerMed)^1^ emphasizes the need for collaborative efforts across various fields, including biomedical, social, and economic sciences coupled to technological advancements. Among these technological advancements, nanocarrier (NC), such as lipid-based carriers or polymeric based nanoparticles, have emerged as a promising avenue to achieve the goals of personalized medicine. They can be tailored on a molecular level to target pathogenic mechanisms and be composed of specifically suited materials to match a disease profile. NC size and shape can be harnessed to achieve customizable treatments to fulfill an individual application. However, due to numerous biological processes occurring on the nanoscale, clinical translation of nanomedical strategies requires a comprehensive understanding of NC’s interactions within their biological context, both during transport in blood, and in complex tissues and cellular environments. To bridge this gap, the development of enhanced model systems becomes essential as currently used methodologies including static cell culture setups and animal models have fallen short in providing accurate information on NC’s biological behavior due to their misrepresentation of human physiology. This Review outlines novel model systems as approaches to explore the capacity of nanomedicines to facilitate individualized therapies (Figure 1).

Upon administration and adsorption into the bloodstream, traditional treatments, such as small molecules, disperse freely throughout the body. In addition to achieving their intended effects, their therapeutic shortcomings can include introducing toxic side effects due to challenges in targeting specific sites.^2^ In the realm of personalized medicine, such non-targeted therapeutics fall short of their potential, where more sophisticated results can be achieved using NCs. Their nanoscale size enables them to navigate biological barriers, achieve high tissue uptake, increase circulation, and interact with specific cellular receptors or molecules.^2−4^ Their medical applications have significantly expanded in recent years,^5^ where some examples include crossing the blood-brain barrier for neurological disorders, navigating dense tumor tissues, and even facilitating drug delivery to previously inaccessible chondrocytes in the cartilage, which we and others have shown.^6,7^

## Nanocarriers as Precision Delivery Vehicles for Personalized Medicine

**Figure 2:**
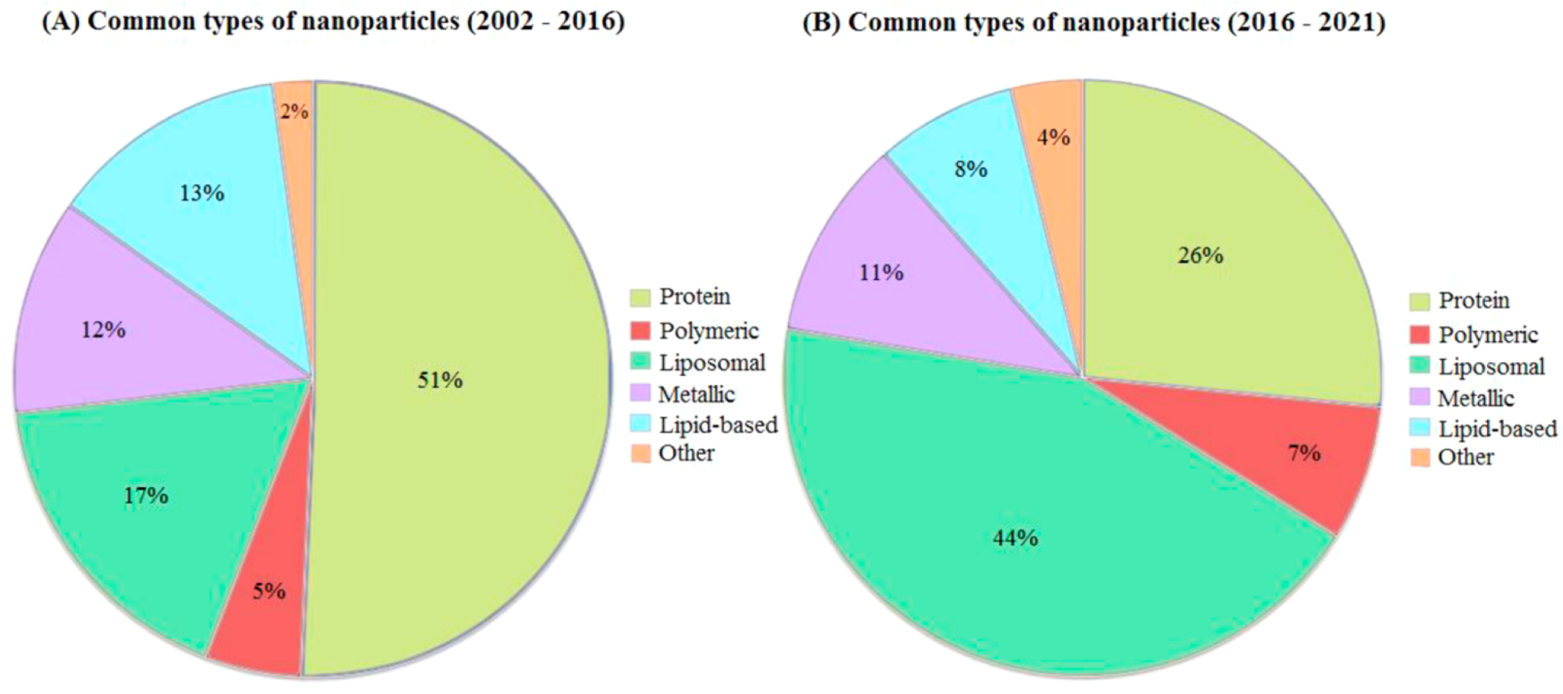
Common types of nanoparticles. This figure contains information about the types of nanoparticles used in clinical trials. (**A**) Pie chart A represents the types of nanoparticles in clinical trials from 2002 to 2016. The group of other consists of carbon-based, silica-based nanoparticles, and nanostructured formulations of hormones. In the 2002–2016 period, the most abundant type of nanoparticles in clinical trials was protein (51%). Liposomal formulations were the second most common but were still relatively low (17%). Lipid-based nanoparticles could be encountered in 13% of all clinical trials during that period. Both metallic and polymeric formulations appeared to be scarce (12 and 5% respectively). (**B**) This part of the figure represents types of nanoparticles in trials from 2016 to 2021. The other group consists of quantum dots, micellar nanoparticles, and exosomes. In comparison to 2002–2016, protein nanoparticles demonstrated a downfall (from 51% to 26%) which can be explained by an increase in liposomal drugs (from 17% to 44%). Lipid-based formulations also faced a slight decrease to 8%, while metallic and polymeric drug percentages stayed almost the same (11% and 7%, respectively). Reprinted with permission under a Creative Commons CC BY 4.0 license from ref (22). Copyright 2023 MDPI.

NCs are highly adaptable from both a material composition and an encapsulation perspective. NCs can be composed of many different materials, including lipid carriers such as the COVID-19 vaccines,^8^ polymers,^9^ dendrimers,^10^ or mesoporous silica.^11^ These materials are highly diverse, but all offer adaptability in their physical properties such as size and attachment of targeting moieties. They are adaptable toward biological challenges and are diverse in their cargo carrying capacity. NCs can also enable controlled and sustained release of therapeutic agents over time, maintaining therapeutic levels while reducing administration frequency.^12^ We and other groups have previously shown that employing nano-, and micron sized carriers consisting of responsive materials to the diseased microenvironment for drug release enable the use of lower drug quantities.^13−15^ With their capability to encapsulate a diverse array of therapeutic agents, ranging from small molecules and proteins to nucleic acids and combinations of drugs, NC adaptability allows for treatments tailored to individual needs. Their ability to carry potent therapeutic agents with poor solubility and high toxicity has long been recognized in the form of Doxil, the first clinically approved nanodrug.^16^ In addition, NCs can be equipped with imaging agents or sensors, allowing for real-time monitoring of drug distribution, release, and therapeutic effects.^17^ This becomes particularly pertinent in the context of personalized medicine, where treatments should be tailored to an individual’s unique disease characteristics and response to therapy. Yet, NCs’ adaptability and inherent flexibility in composition, architecture, and therapeutic cargo contribute to their challenge in clinical translation.^18^ In developing efficient NCs, there can be issues with loading, stability, and reproducibility, to name a few.^19^ The interactions between NCs, biological systems, and intricate disease pathways are incredibly complex, demanding a level of precision that mandatory animal models struggle to achieve.^19^ Wilhelm et al. compiled data from clinical studies revealing that only <1% of administered NC dose reached the intended targeted solid tumors.^20^ While the number of clinically available nanomedicines is increasing, they are still below the projections for the field as they face additional challenges compared to conventional drug development.^5,17,19,21,22^ Since 2016, lipid-based and liposomal nanoparticles have been the most common in both clinical trials (Figure 2) and FDA-approved drugs.^22^ In 2021, over 30 nanoparticles have been approved and used in clinical applications.^23^

However, not even choosing the most suitable animal model may lead to higher predictability in clinical trials. Compared to conventional drugs, nanomedicines that have been tested on animals appear to result in less predictable outcomes.^24,25^ This may be attributed to nanomedicine’s effectiveness being heavily reliant on factors such as transport, target site accumulation and penetration, drug release at the target site, and tissue distribution. These differ significantly between animals and humans, where NCs are subjected to laminar flow, collisional, and binding interactions with blood cells and interactions with serum proteins.^26−30^ To truly harness the potential of nanomedicine, specifically in the context of personalized medicine, there is a clear need for more advanced model systems tailored to the intricacies of nanoscale interactions. Traditional *in vitro* cellular assays have been instrumental in studying NC delivery mechanisms and enabling large-scale NC screens. While their advantages include being inexpensive, well established, ease of readout and with a plethora of scientific literature to compare results to, their lack of 3D-architecture, cell–cell and cell-matrix interactions, and with no mechanical forces or gradients, they fail to capture intricate biological dynamic processess.^31,32^ Animal studies, mainly reliant on mice, better recapitulate the *in vivo* setting and have enabled researchers to determine NC circulation times, biodistribution, and efficacy. However, the misrepresentation of human anatomy and physiology has led to inconsistencies between animal data and clinical trial data, and intensifies the call to reduce animal testing and develop better and more clinically representative models.^30,33−36^ Recently, the obligation for animal testing has experienced an alleviation with the introduction of the Federal Food and Drug Administration (FDA) Modernization Act 2.0 in 2022,^37^ which encourages the use of novel model systems to help in evaluating mechanisms resulting in enhanced translatability. These models must emulate the dynamic behavior of NCs within biological environments in general, accounting for factors such as physiological differences in blood vessels, tissue thickness and composite, cellular uptake machineries, and potential toxicities. Novel technologies like microfluidic platforms, 3D tissue constructs, and computational simulations can help fill this gap and can be adaptable to personalized medicine approaches by incorporating patient-derived material.

## Advanced Models to Explore Personalized Nanomedicines

**Figure 3:**
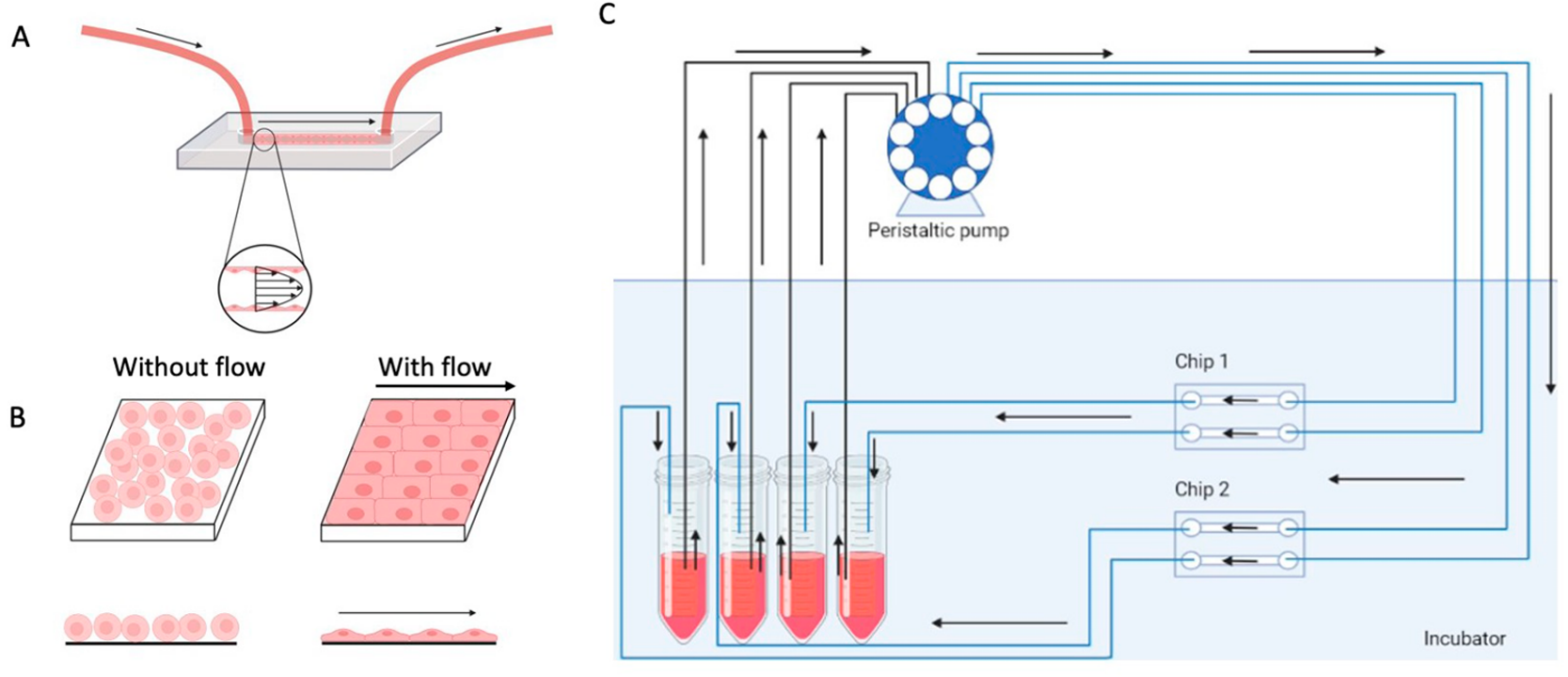
Endothelial cell cultivation within a microfluidic chip under dynamic flow conditions (A). Continuous perfusion subjects the endothelial cells to shear stress, resulting in cellular elongation aligned with the flow direction, mimicking a tissue structure closer to that under *in vivo* conditions (B). Example of a complete microfluidic setup enabling parallelly coupled perfusion experiments on cells cultured within the microfluidic channels (C). Created with BioRender.com

The overall goal of advanced model systems is to accurately represent human physiology and provide an increased understanding of biological phenomena that plays a role in medicine and pharmacology. To accurately replicate the complex interactions between NCs, human physiology and intricate pathological environments, novel techniques aim to mimic key organotypic cells and functions, emulate the extracellular matrix (ECM), biophysical cues, and include biochemical factors that make up physiological aspects of the tissue. Manipulation of flow rates existing in blood vessels can be added, pressure can be applied, as well as adjusting oxygen and pH levels which results in controlled culture conditions. They offer new paths for exploring and modeling our most common diseases such as cancer, atherosclerosis, and inflammation, but can also uncover information regarding more rare pathogenic events and opens excellent possibilities to explore mechanisms of rare diseases. Microfluidic platforms such as organ-on-a-chip technology represents such a potential approach by their ability to include human-derived tissue models within microfluidic chips.^30^ Leveraging tissue engineering, microfluidics, and advanced cell culture, this technology creates tailored cellular environments, including fluidic, mechanical, and structural control.^30^ A subset of this technology is vessel-on-a-chip (VoC) systems, specifically designed to mimic human blood vessels *in vitro* (Figure 3). These models can help nanomedical designers to evaluate the obstacles encountered by the particles in blood, and how they are influenced by physical phenomena such as shear stress.

More specialized compartments in the body, such as the joints that play a pivotal role in the overall function and movement of the human body, also demand more sophisticated delivery technologies and model systems.^38^ Insights into how personalized nanomedicines behave in such compartments require development of models that accurately represent their structure, mechanics, and behavior.^38^ To achieve a comprehensive understanding of joint biology and pathology, a combination of approaches is necessary, especially when designing nanomedicines for these compartments. This Review paper covers aspects of the models listed in Table 1.

**Table 1:** Advanced Models for NP Characterization in Blood and Joints

**Bloodstream Extravasation Models**	
Vessel-on-a-Chip	NC transport across endothelial barrier
**Joint Models**	
Computational models	Tissue mechanics, function, and NC toxicity
*Ex vivo* explant model	NC transport and mechanistic interactions in tissue-specific environment
3D bioprinting	Fabrication of complex and biomimetic joint tissue structures
Bioreactor systems	Assessment of NC efficacy in physiologically relevant conditions
Combining 3D bioprinting and bioreactors	Assessment of NC in complex biomimetic and physiologically relevant conditions

### Vessel-on-a-Chip Models: Understanding Nanomedicine Transport

**Figure 4:**
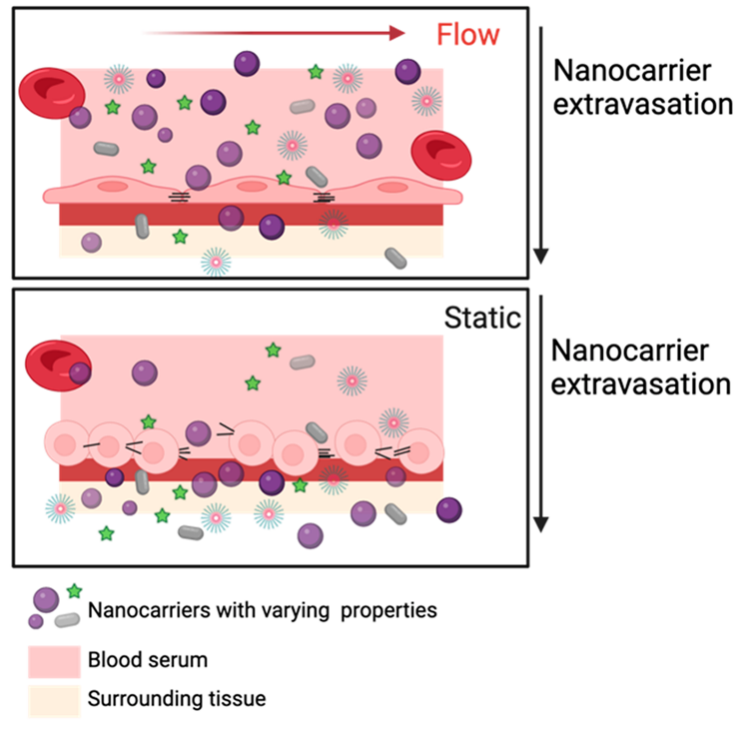
Illustration of nanocarrier extravasation through the endothelial barrier, where the permeability of the barrier is influenced by the integrity of the cellular junctions. Created with BioRender.com.

A key step for NCs to effectively reach their intended target organs is their successful extravasation out of the bloodstream. When NCs are administered via the intravascular route, endothelial cells (EC) represent the first cells with which they interact to reach the target tissues. These cells control the permeability across the blood vessel walls. Conventional models of cell culture experiments and transwell systems are hampered by their static conditions, limiting their ability to mimic the complex physiological environment. In a recent study, Gimondi et al. compared NC’s capacity to traverse the endothelial cell lining using either a static transwell *in vitro* model or a VoC model. Their results suggested a higher transport rate of NCs upon administration for the static condition which could be due to an accumulation at the targeted site, as opposed to the dynamic counterpart.^39^ By introducing dynamic flow and shear stress conditions akin to those in the human body, the VoC model enables the replication of physiological parameters that influence NC interactions.^31,40,41^ The permeability of ECs, one of the main functions of the inner endothelial cell layer, is affected by the integrity of the cell junctions. Applying flow to ECs can have a significant effect and in order to resemble the *in vivo* EC layer, it is important to culture the cells under flow (Figure 3B).^42^ The shear stress stimulus significantly affects cell adhesion properties and behavior, leading to ion channel activation, gene expression modification, and cellular layer reorganization, ultimately impacting permeability.^43^ This event has also been shown to significantly shape cellular interactions with NCs.^44^ NC extravasation across the ECs occurs through two pathways: paracellular and transcellular. In paracellular extravasation, NCs passively exit the vascular lumen via gaps in the endothelium, while transcellular pathways involve passage through the cellular membrane (Figure 4).^45^ In addition, ECs respond to various chemical and physical stimuli, and control processes such as hemostasis, vasomotor tone, immune responses, and inflammation.^46,47^ Growth factors, cytokines, oxygen, and mechanical stress play important roles in contributing to the heterogeneity of the ECs.^44^

**Figure 5:**
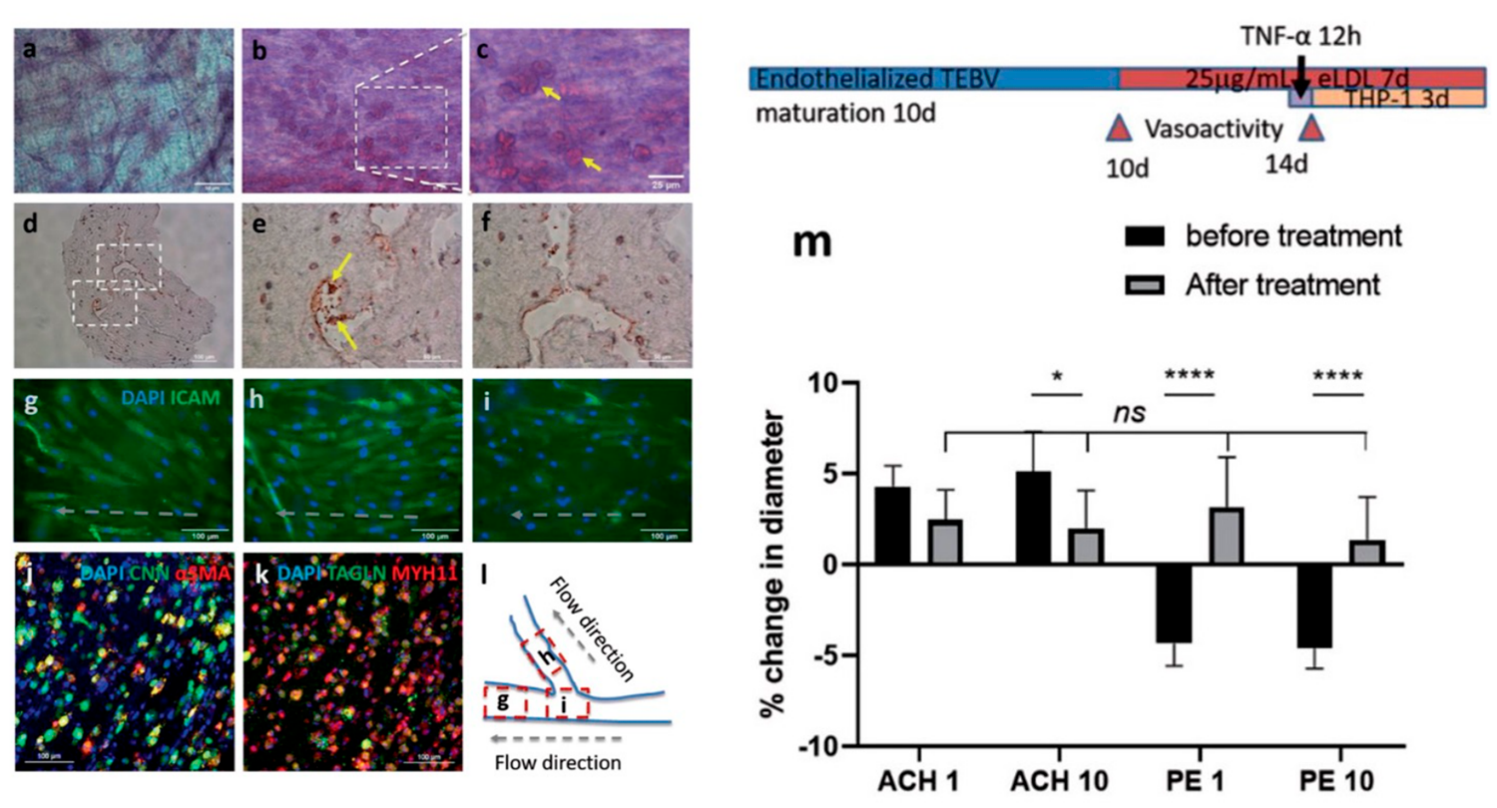
TEBV function and the effect of eLDL/TNF-α treatment. Oil-red-O staining of lipid-laden macrophages (foam cells) in brTEBVs without (a) and with (b–f) eLDL and TNF-α treatment: (a–c) tissue staining, (d–f) staining of frozen sections. (g–i) Immunofluorescence images of endothelial layer in brTEBV tissue, with ICAM-1 (green) and DAPI (blue), after 5 days of culture and 72 h treatment of 10 μg mL^–1^ eLDL and 8 h TNF-α at main (g), side (h), and center (i.e., branching area) (i). (l) Schematic diagram indicating the location of each image and direction of flow in (g)–(i). (j,k) Confocal immunofluorescence images of TEBV tissues after the treatment; (j) Calponin (CNN, green) and α-SMA (red); (k) TAGLN (green) and MYH11 (red). Colocalization of CNN and α-SMA, and of TAGLN and MYH11 is indicated by yellow fluorescence. (m) The change in vasoconstriction and dilation capabilities in linear TEBVs induced by 1 or 10  ×  10^–6^ m acetylcholine (Ach) and phenylephrine (PE), before and after 96 h treatment with eLDL and TNF-α treatment; *n* = 6 (3 locations/vessel, 2 vessels/condition), two-way ANOVA multiple comparisons. *P < 0.05, ****P < 0.0001. Scale bars: 100 μm (d,g–k), 50 μm (a,b,e,f), 25 μm (c). Panels (a)–(k) are from brTEBVs and panel (m) is from linear TEBV. Adapted from ref (48). Copyright 2021 John Wiley and Sons.

The VoC therefore represents an excellent model for evaluating personalized medicines as suitable biomechanical cues and/or molecular mechanisms are essential to mimic the specific desired conditions. For instance, Lee et al. designed a chip to mimic the characteristics of blood vessels in atherosclerosis or thrombosis (Figure 5).^48^ They developed tissue engineered blood vessel (TEBV) technology to model the processes of early atherosclerosis by introducing branched TEBVs (brTEBVs). They tested various angles to recapitulate bifurcation areas of coronary arterioles and induced EC dysfunction and monocyte adhesion by stimulating the ECs either enzyme-modified low-density lipoprotein (eLDL) or tumor necrosis factor alpha (TNF-α). Such disease-specific models allow for the evaluation of therapeutic efficacy by combining genetic insights with microfluidic technology.

By adjustment of the VoC model to specific biomarkers and disease mechanisms, it becomes possible to design experiments that simulate how an individual’s blood vessels will respond to different nanomedical interventions. The VoC can directly be integrated with patient-derived sera and cells, creating a microenvironment that closely resembles the individual’s physiology.^49^ Importantly, studying the formation of a protein corona (PC) on NCs could yield detailed information on how to optimize the materials and size composition that best fits an individual’s physiology. The formation of a PC, a coating of proteins, and other biomolecules surrounding the NPs, grants them a distinct biological identity that influence the treatment outcomes.^50,51^ Ju et al. demonstrated how this phenomena is highly specific for each donor and individual, and how the varied PC dictated the interactions with immune cells.^52^ This phenomenon occurred regardless of the material and size of the NCs, suggesting the importance of studying the PC to optimize the NC for each individual. Given that the clearance of NCs by the immune system poses a significant obstacle to overcome for the efficiency of NCs, understanding NC interactions with the immune system is pivotal for the success of nanomedicines, and using VoCs with integrated patient materials could give insights into these phenomena. We have previously demonstrated the hampering effect of the PC formed from arthritic patient synovial fluids on the uptake of positively charged NPs into cartilage explants and joint-related cells.^6^ The PCs were disease specific, and subsequent coated NC uptake differed both in cells and in tissue from the standard fetal calf serum that many researchers use, emphasizing the need to integrate specific pathogenic biomarkers and environments into NC research.

### Specialized Compartments: Advanced Tissue Models for Joint-Related Diseases

In general, achieving effective drug uptake and retention within specialized tissues poses a formidable challenge. An example of the need for new model systems to develop personalized medicine approaches will be represented here by the need for developing enhanced treatment strategies for osteoarthritis (OA). There are multiple types of joint diseases beyond the scope of this review; this paper only addresses solutions for OA. OA is the most prevalent joint disorder and affects more than 240 million people globally. It is a debilitating disease characterized by cartilage degeneration, accompanied by mild local inflammation.^53^ This erosion of joint cartilage leads to subchondral bone damage, increased pain sensitivity, and synovial inflammation. In the context of OA, a disability that currently is without treatment options,^54^ intricate challenges arise due to the presence of diverse patient subgroups and multifaceted disease etiologies.^55^ Additionally, obstacles in delivering drugs to OA patients include the rapid clearance of therapeutic agents from joint spaces and the difficulty in penetrating dense cartilage tissue. The cartilage ECM of the tissue comprises tightly packed proteoglycans with strong negative charges, intricately intertwined within a complex collagen network.^56,57^ Overcoming these challenges necessitates the development of advanced drug delivery systems such as NCs that can enhance the targeting and efficacy of treatments.^38,58^ From a materials and targeting perspective, several strategies have emerged such as micron sized particles to remain in the joint space, or cationic small particles that are able to enter the dense ECM.^38^ However, the conventionally used small animal models inappropriately represent human cartilage tissue depth, and 2D and 3D cell models have issues with replicating the complex biological tissue structures.^38^ Therefore, developing treatment strategies for OA can mainly be achieved by using a variety of advanced models beyond the conventional *in vitro* setting, presenting an opportunity to integrate personalized medicine approaches. This includes utilizing computational rendering, tissue *ex vivo* explants, 3D bioprinting, as well as bioreactors.

**Figure 6:**
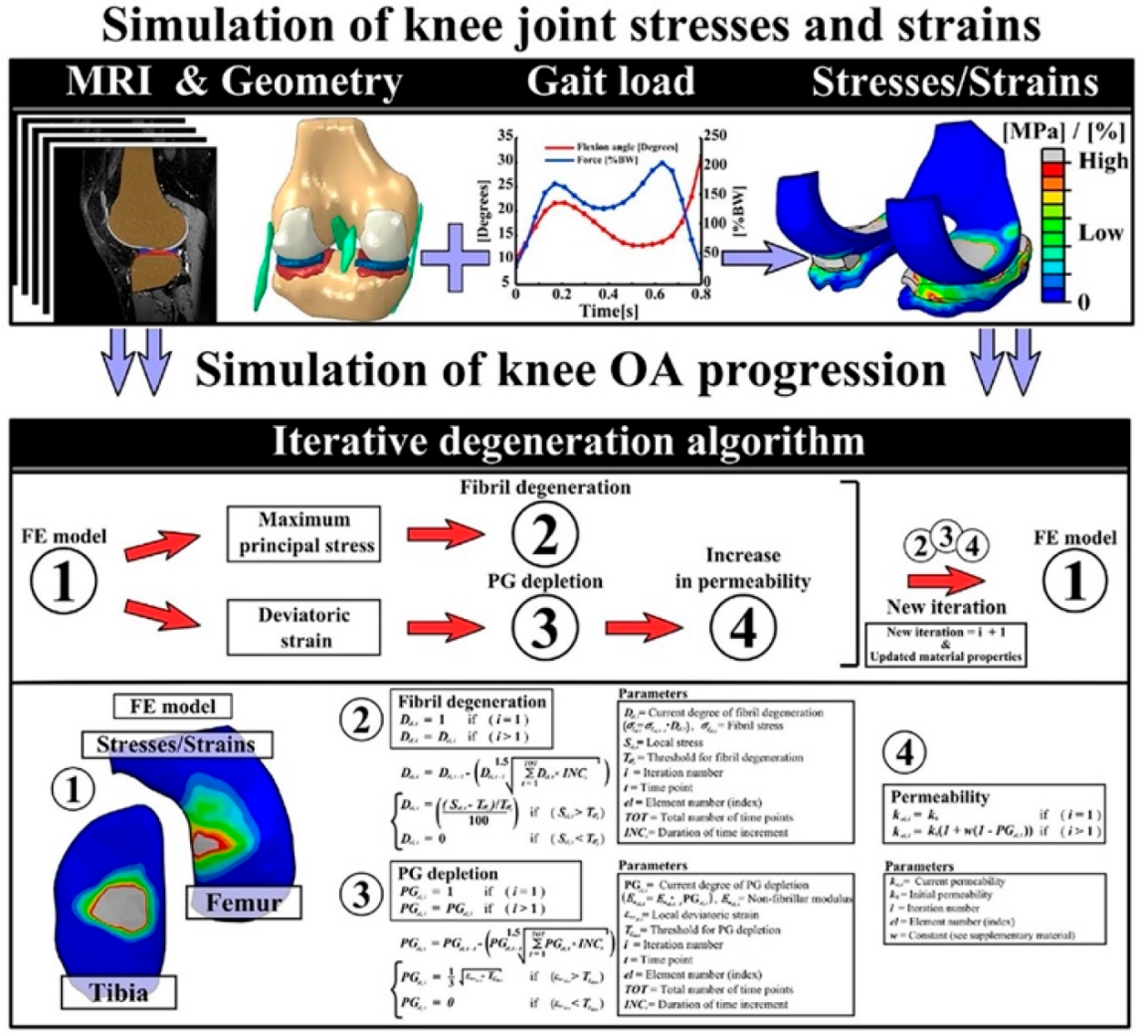
Workflow from geometry creation to the iterative degeneration algorithm. The top row describes interphases which are considered when calculating tissue stresses and strains, whereas the bottom row presents how predicted stresses and strains affect (1) collagen fibril degeneration (2), proteoglycan depletion (3) and increase of permeability (4) in the algorithm. Reproduced from ref (69). Copyright 2018 John Wiley and Sons.

**Computational models** are useful for multivariate screening purposes that can be subsequently validated in live experiments. Based on mathematical equations and computer-driven analysis, models such as finite element analysis (FEA) or multibody dynamic simulations can provide valuable insights into tissue mechanics, function, and NC toxicity.^59,60^ They offer a cost-effective approach to study joint biomechanics and allow rapid exploration of different scenarios and parameter variations. Several studies have addressed joint behavior under various conditions including loading, lubrication, and mechanics of the cartilage and surrounding tissues.^61,62^ However, they rely on accurate input data and assumptions, which may introduce uncertainties and simplifications.^63,64^ For example, while there are numerous studies addressing inorganic or carbon-based NC transport, polymeric NC models are more difficult to build due to more complex structural properties and more sensitive physicochemical parameters.^65^ The complexity of the NC-cell interactions also poses an additional challenge where a vast majority of descriptors must be included to replicate the dynamic nature of cellular membranes and the glycocalyx.^66,67^ Such drawbacks may make computational models generalized or biased, missing out on biologically relevant complexities of *in vivo* joint physiology. As artificial intelligence is on the rise, the prospect of computational models getting more advanced is evident. Advancements in medical imaging techniques now allow patient-specific tissue models to be incorporated into treatment strategies. Mononen et al. combined MRI and computational modeling, enabling the prediction and evaluation of OA progression in response to specific loading conditions for individual patients (Figure 6).^68,69^ By incorporation of patient-specific anatomy and biomechanics, these models enable personalized simulations and preoperative planning for surgeries. However, it is important to note that the computational model development and improvement rely on validation against experimental data to ensure accuracy and reliability.

**Figure 7:**
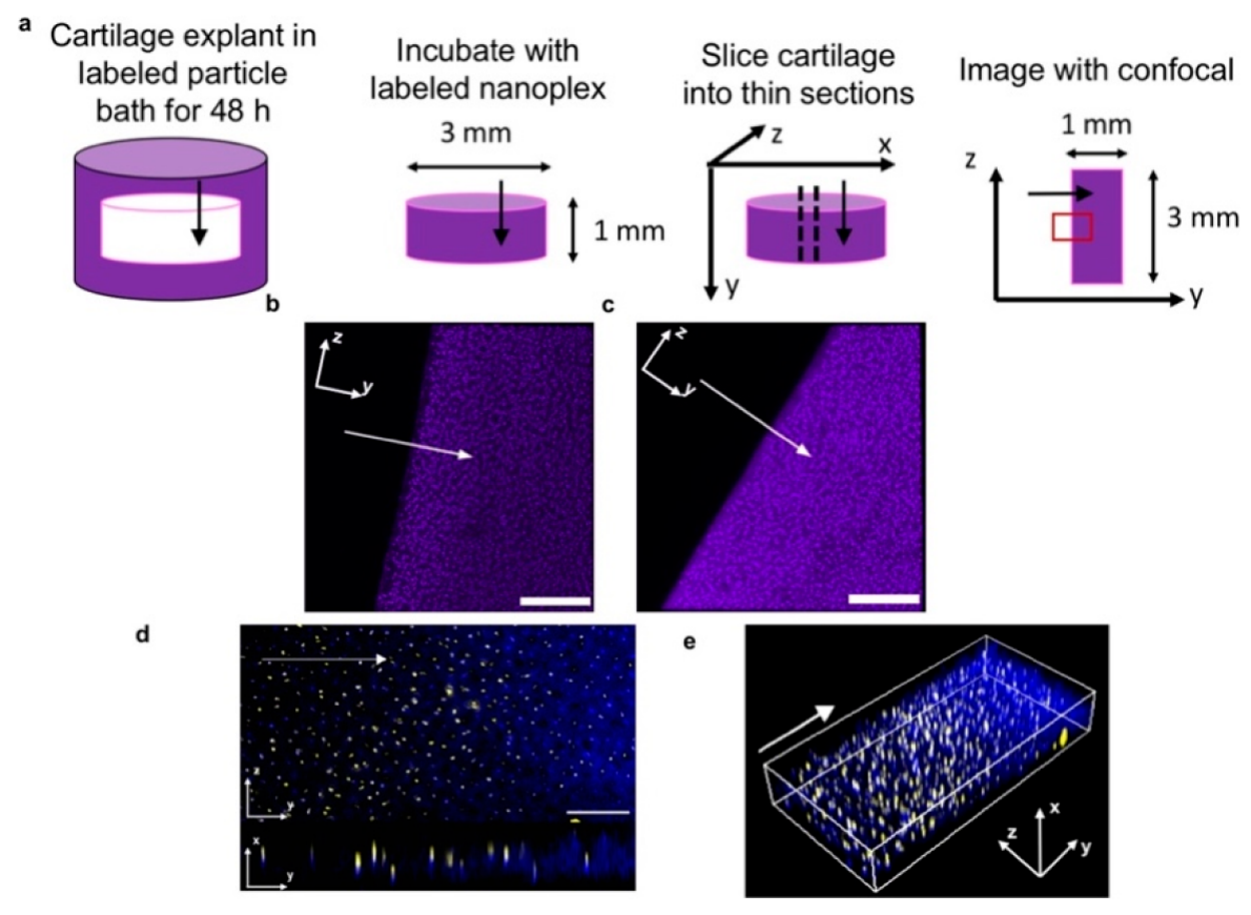
Nanoplex penetration into bovine cartilage explants. Arrows indicate the direction of diffusion. Cartilage culture media consisted of DMEM with added FBS, nonessential amino acids, sodium pyruvate, HEPES, ascorbate, and proline unless otherwise stated. Particle treatment occurred in PBS. (a) Schematic for visualizing diffusion gradients of fluorescently labeled IGF-1 (purple) within the cartilage tissue. The red box indicates field of view shown. (b) Nanoplex delivered IGF-1 and (c) IGF-1 alone distributed throughout the tissue (Scale bar = 200 μm) (d) Penetration of nanoplex components (yellow: polyArg, blue: IGF-1) throughout the inflamed cartilage tissue. Inflammation in cartilage tissue was simulated by the addition of 10 ng/mL of IL-1α to cartilage media. Top: *yz* view; bottom: *yx* view. Scale bar = 100 μm. (e) 3D visualization of panel (d). Reprinted with permission under a Creative Commons CC BY 4.0 license from ref (73). Copyright 2016 American Institute of Chemical Engineers.

One such validation model is the ***ex vivo*****explant model**, where intact joint tissue or organs are extracted and maintained in a culture system outside of the body.^70,71^ This technique preserves the native architecture and cellular interactions of the joint tissues, allowing a direct assessment of tissue responses to drug treatments or mechanical stimuli on tissue regeneration or degradation (Figure 7).^73^ Joint tissue explants may be acquired from many different animal models, with the most prominent being the porcine, equine, bovine, and human tissue sources. These tissues can serve as direct models for individual patients to achieve personalized medicine, although ethical concerns arise regarding tissue procurement, particularly in the context of human samples. Cartilage lacks blood vessels and innervations, thus requiring additional considerations for NC design. We and others have used explant models to study NC transport and mechanistic interactions in tissue-specific environments.^4,72,73^ This allowed us to assess the NC behavior in a relatively native yet controlled setting. While it offers a simplified and cost-effective approach to investigating joint biology, the finite lifespan of excised tissues can hinder long-term experiments.

**Figure 8:**
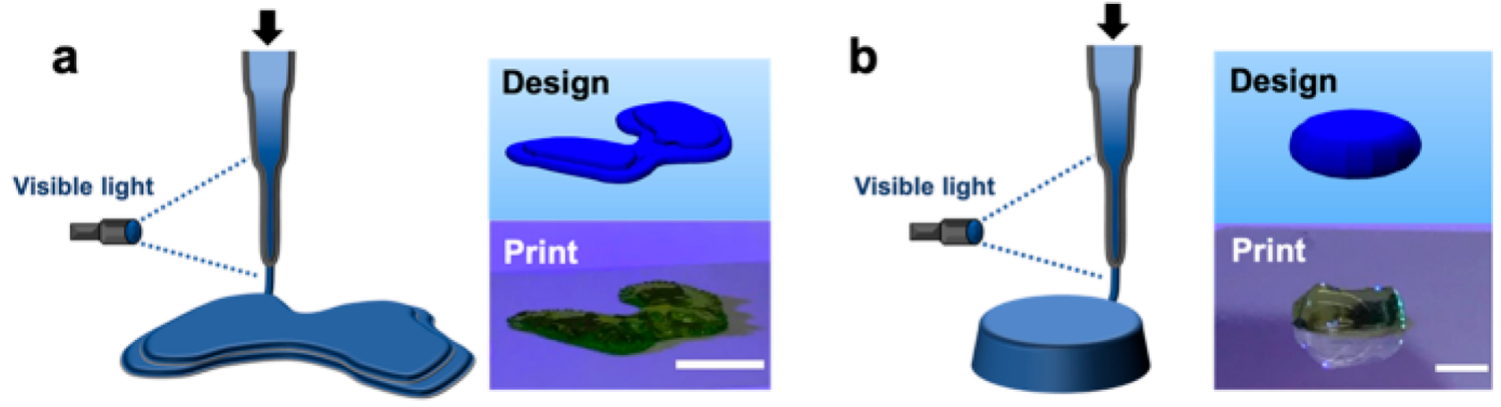
Representative multilayered constructs printed via *in situ* cross-linking. Left: Schematic of *in situ* cross-linking method and Right: CAD design and representative image of a printed construct (labeled with food coloring) for designs of (a) a model femoral condyle or (b) a disc (∼1.5 mm thickness and ∼6.5 mm diameter). Scale bars: 1 cm (a) and 5 mm (b). Reprinted with permission under a Creative Commons CC BY 4.0 license from ref (78). Copyright 2019 Springer Nature.

A more tailored and adjustable tissue model can be achieved by using **3D bioprinting** to create constructs that mimic the architecture and composition of joint tissues. It involves the fabrication of three-dimensional structures using biocompatible materials and living cells.^74−77^ They enable the fabrication of complex and biomimetic joint tissue structures, provide a platform for studying tissue development, disease progression, and therapeutic interventions, allow customization and control over tissue composition (Figure 8).^78^ Current bioprinting techniques are relatively expensive and pose limitations in replicating the complexity and functionality of native joint tissues, where challenges include achieving vascularization and innervation. Thus, 3D tissue bioprinting has largely focused on tissues like cartilage due to the lack of blood vessels and nerves. Hydrogel-based strategies have risen to popularity as they allow easy incorporation of various biological factors and even NCs that serve as therapeutic depots as the 3D printed scaffolds integrate with the tissue.^79^ Incorporation of NCs into 3D materials is a promising composite approach, with important implications for personalized regenerative medicine.

Besides 3D bioprinting, **bioreactor systems** have been employed for a long time to mimic the mechanical and biochemical environments of joints. They typically consist of cells or tissues cultured in a controlled environment, allowing researchers to study various aspects of joint physiology and pathology.^80^ Bioreactors can be used to investigate multiple effects on a tissue at once and allow more variation than other wet-lab counterparts. By implementing tissues and fluids from patients, these models can be used to stratify patient-specific disease mechanisms to develop novel therapies and evaluate candidate biomarkers. Bioreactors allow for more realistic physiological environment compared to traditional cell culture as they allow precise control of mechanical and biochemical factors (loading conditions, nutrient supply, and growth factors), thus permitting a more extensive assessment of NC efficacy in physiologically relevant conditions.^81^ While full customization and easily integrated patient-derived material make them highly useful in the context of personalized medicine, bioreactors can be technically complex and require specialized equipment and expertise, thus hindering the equivalency of the *in vivo* environment of a joint.

**Figure 9:**
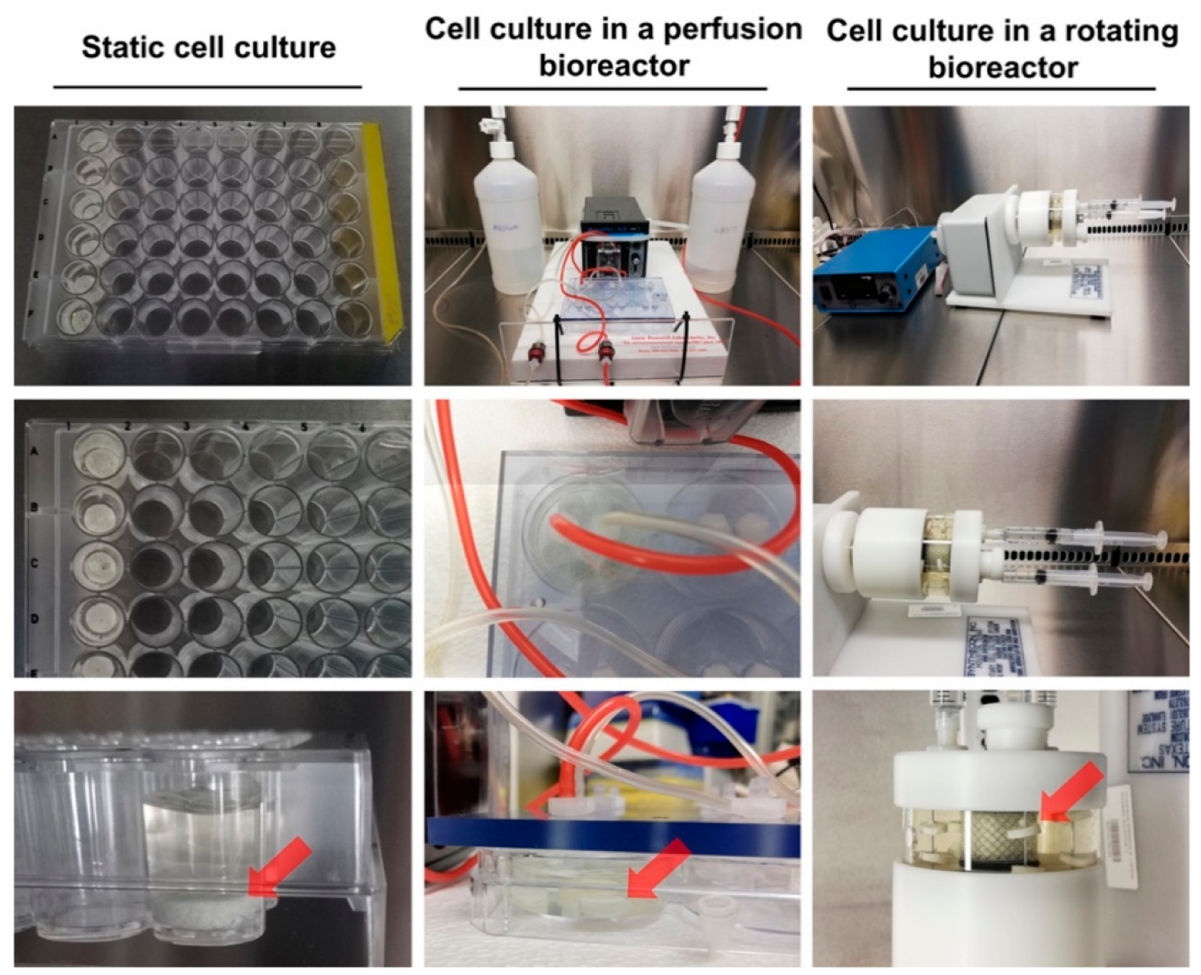
Setup of 3D BMDSC cultures in static conditions, in the perfusion bioreactor (Lazar Arrow-MTM Micro Bioreactor System), and in the rotating bioreactor (Rotary Cell Culture System (RCCS), Synthecon). Red arrows indicate the placement of cell-seeded biomaterials. Reprinted with permission under a Creative Commons CC BY 4.0 license from ref (80). Copyright 2023 Springer Nature.

In the future, a combination of advanced models and techniques is most likely needed. Utilizing composite techniques such as 3D printing and perfused bioreactors, Forrestal et al. engineered personalized implants capable of long-term tissue growth and enhanced nutrient transport.^82^ Kazimierczak et al. further compared the effectiveness of rotating and perfusion bioreactors in the production of a living bone construct using human bone marrow-derived mesenchymal stem cells (BMDSCs) (Figure 9).^80^ These studies demonstrate that the most efficient outcomes may arise from the combination of different technologies, thus resulting in more complex and robust testing systems, further emphasising cross-disciplinary collaborations to drive the innovation process.

## Challenges and Opportunities in Personalized Nanomedicine

Due to genetic and microenvironmental heterogeneities, patients’ responses to drugs can exhibit significant variability, underscoring the need for accurate evaluation of therapeutic efficacy and individualized optimization. While efficient they often lack precision and inadvertently affect nontargeted elements. This can result in undesired collateral effects on various physiological system. The scenario of OA as described earlier exemplifies a condition characterized by intricate disease origins, patient-specific variables, and considerable patient suffering, all of which emphasize the urgent need for tailored and personalized therapeutic approaches based on NC technology. However, recognizing that diseases often manifest in multifarious ways across individuals, NCs customized to each patient’s unique disease profile is a complex undertaking. Designing NCs that accurately address the distinct aspects of a patient’s illness requires navigating the complexities of disease progression, molecular mechanisms, and therapeutic targets.

However, the process of developing new model systems is not only complex and time-intensive but also financially demanding, requiring substantial investments. Prior to the realization of personalized therapies, a critical prerequisite involves establishing standardized, cost-effective models that can be produced on a large scale. This groundwork is essential to pave the way for the eventual integration of personalized therapies into clinical practice. Developing enhanced treatment strategies based on specific patient demographics, disease classifications, and even gender-related factors can be an initial step toward precision medicine, while not yet personalized. Acknowledgment of gender-specific variations in disease susceptibility, progression, and responses to treatment is steadily growing. A pertinent example lies in cardiovascular diseases, where disparities between men and women driven by hormonal influences, can lead to distinct clinical manifestations.^83^ In this context, NCs emerge as valuable tools to facilitate the delivery of treatments to meet these gender-specific intricacies.^84^

A critical aspect of personalized medicine include ethical considerations and the issue of privacy. As healthcare systems collect and analyze patient-specific data to tailor treatments, questions about the privacy and security of this sensitive information emerges. Central to this discourse is the concept of data security. When personal data are accessed, stored, and used to craft individualized treatment plans, it inherently raises concerns about unauthorized access, data breaches, and the potential misuse of personal information. These concerns are particularly pertinent given the highly sensitive nature of medical and genetic data. Patients have the right to be informed about the collection, storage, and utilization of their data for personalized treatment purposes. Providing clear, comprehensible information and obtaining explicit consent from patients have become ethical imperatives in safeguarding their autonomy and ensuring that they actively participate in decisions regarding their healthcare. The challenge involves a delicate balance between utilizing patient data to optimize treatment outcomes and preserving patient privacy. Achieving this equilibrium necessitates robust data protection measures, stringent security protocols, and comprehensive regulations that dictate how patient information is managed and shared. Additionally, it demands transparent communication with patients, ensuring that they comprehend the implications of sharing their data and the potential benefits they stand to gain from personalized treatments. The multifaceted nature of this challenge extends beyond the realm of ethics and patient welfare; it also intersects with legal and regulatory frameworks. Laws pertaining to data privacy and healthcare, such as the General Data Protection Regulation (GDPR) in Europe, contribute to the landscape of personalized medicine by aiding stringent requirements on data handling and patient consent.

Currently developed model systems rely on allogeneic cells to construct their synthetic platforms, such as bioreactors and organs-on-a-chip. While not patient specific, these processes have illustrated several technical issues facing these advanced models. Often developed in-house, the scientific community has recognized the need for standardized frameworks that can yield reproducible outcomes. This challenge increases when one aims to devise personalized systems that incorporate patient-specific cells. Significant hurdles regarding limitations in obtaining patient material such as potential invasive sample collection, scarcity, and unreliability of patient cell sources, as well as limitations due to low proliferative potential of the material. Undertaking this endeavor mandates considerable technical expertise and rigorous clinical validation.

In conclusion, personalized treatments that leverage nanomedicines hold the key to addressing the diverse and intricate complexities of various diseases ranging from cancer to cardiovascular disorders to neurodegenerative conditions. By aligning the potential of nanomedicine with advanced model systems, personalized medicine can improve traditional approaches and allow for precision healthcare, where treatments are as unique as the individuals receiving them. Merging nanomedicine with innovative model systems could be a path to unlocking the full potential of personalized nanomedicine and to shape the future of healthcare.
